# Tales the double helix tells

**DOI:** 10.1186/2041-2223-1-2

**Published:** 2010-09-01

**Authors:** Mark A Jobling

**Affiliations:** 1 Department of Genetics, University of Leicester, Leicester, UK

## 

When Manfred Kayser, one of the Editors-in-Chief of this new journal, asked me to write a regular column for *Investigative Genetics*, I declined. We all have too much to do; I'm supposed to be writing the second edition of a textbook in a field, human evolutionary genetics, in which major papers appear with a frequency that ought to be exhilarating, but turns out to be depressing, each of them requiring major textual upheavals, and prompting us authors to wish for a moratorium on research for a couple of years to allow us to catch up. But, having agreed to do it (Manfred was persistent) the worst was to come. He held up as a model the regular 'Comment' columns of Greg Petsko in *Genome Biology *[1], and when a collection of Petsko pieces arrived in my inbox, I realised what I had to live up to--erudite, elegant, wide-ranging, witty--hell, he even has a couple of dogs who occasionally write his column for him (making his surname strangely appropriate). All I can muster in my household is an idle and decidedly non-literate cat, a crested gecko and a variety of fish. No help there, then. I hope in the future to attract guest writers, and I invite suggestions for topics of interest from readers.

And so, to work. This new journal is about DNA. Not so much about what DNA does and how it does it, but more about the tales it can tell. These are tales of relationships, or origins, or what someone looks like, or whether they will get sick or not. And just as story-telling has evolved from the campfire performance to the book, the movie, radio, TV, the blog, the e-book and the tweet, DNA's story-telling is evolving rapidly too, under the influence of technological advance, commercialisation, the Internet, politics and a large dose of wishful thinking. For this reason, now is a particularly interesting time for a journal like this to establish itself.

Forensic geneticists have traditionally dealt in relatively simple tales--this person left their blood here, or this man is this child's father. With the introduction of familial testing, phenotype prediction, and population-of-origin estimation, the tales are becoming more embellished. There's a danger that they could enter the realm of fantasy; a disturbing example was the 'Human Provenance Project' launched as a pilot scheme last September by the UK Border Agency [2] to deduce the 'nationality' of asylum seekers using stable-isotope and DNA-based methods. The idea that simple DNA markers respect political boundaries, and can distinguish reliably between a Kenyan and a Somalian, is both flawed and dangerous, and the project has gone quiet after a barrage of criticism from scientists and refugee support groups. However, genome-wide methods are becoming cheaper and appropriate databases will grow, so it seems unlikely that these controversial approaches will go away.

Anthropological geneticists have always been story-tellers, but here too there is a shift in emphasis through the rise of personal genetic testing. While something useful can usually be said at the population level, deductions about the origins of individuals, particularly when single loci such as the Y chromosome and mtDNA are used to focus upon single ancestors among many, are perilously insecure; Mark Thomas has called this 'genetic astrology' [3]. And yet for DNA donors, whether eager participants in television programmes, customers of testing companies, or volunteers in academic studies, personal information is what is desired. When one of our DNA donors phones and wants to know if 'he' is a Breton Celt, an Anglo-Saxon, or a Norse Viking, a long and tortuous discussion ensues. Nonetheless, this kind of genealogical testing represents public participation in genetics on a large scale and should be welcomed, provided conclusions are presented honestly.

Current genome-wide approaches to origin stories, using SNP-chips designed for association studies, may type a million markers but to date have yielded little interesting information--showing a Frenchman to be probably French, and a German to be probably German, is hardly revelatory. A recent publication in the *European Journal of Human Genetics *with the striking title 'Genes predict village of origin in rural Europe' [4] appears to provide more promise that with suitable databases, more useful information can be extracted, and this may also rekindle the interest of the UK Border Agency.

When I started out in the 1980s, medical genetics told relatively simple tales too, of genotype-phenotype correlations and causative mutations. With the move towards more common complex traits involving many genetic and environmental factors, today the tale seems more like *Ulysses*. The predictability of such traits from genotypic data is of interest to both forensic and medical communities, and has come into the public arena with the arrival of 'direct-to-consumer' (DTC) genetic testing. Currently, predictability of most traits is lamentably poor, but this has not stopped DTC companies presenting relative risk data to their clients, based on genome- wide scans. The cynic might say that the inclusion of an SNP within the *ABCC11* gene that robustly correlates with ear-wax type makes these tests a very expensive alternative to a cotton-wool bud.

DTC companies have been under particular scrutiny, from the Government Accountability Office in the US, and the Human Genetics Commission in the UK, for example, and recent reports of inconsistencies between the conclusions of different companies and of sample mix-ups must be making life uncomfortable. In this context, it was good to see a scientific paper emerging from one of these companies, 23andMe, in *PLoS Genetics *[5]. The study used genome-wide SNP data on large numbers of customers to seek genetic associations for 22 self-reported traits. Five were pigmentation traits, and reassuringly produced associations that were already known; three traits, hair curling, sneezing in response to a change from dark to light, and the ability to smell a metabolite of asparagus in urine, provided novel associations. Imagine trying to win a grant to study these last three - the idea that interesting biology might ultimately emerge from these commercial activities is cheering.

Over the next few years, each of the areas I have mentioned will be radically transformed as new technologies drive down the cost of whole-genome sequencing, developments that will be covered in the pages of *Investigative Genetics*. It's going to be an interesting ride.
